# Primary cutaneous nocardiosis caused by *Nocardia brasiliensis*: isolation, susceptibility, and molecular identification in eight consecutive cases from a tertiary hospital

**DOI:** 10.3389/fmed.2026.1803484

**Published:** 2026-03-09

**Authors:** Yijin Zhang, Zehu Liu, Xiujiao Xia

**Affiliations:** Department of Dermatology, Hangzhou Third People's Hospital, Hangzhou Third Hospital Affiliated to Zhejiang Chinese Medical University, Hangzhou, China

**Keywords:** diagnosis, epidemiology, infection, *Nocardia brasiliensis*, skin and subcutaneous tissue

## Abstract

Nocardiosis is caused by Gram-positive aerobic bacteria of the genus *Nocardia*, which are soil-dwelling saprophytes. Infection may also occur via direct inoculation, leading to primary infections of the skin and subcutaneous tissues that often manifest as localized nodular lesions. Diagnostic identification can be challenging or even unfeasible using conventional laboratory techniques, particularly when Matrix-Assisted Laser Desorption/Ionization Time-of-Flight Mass Spectrometry (MALDI-TOF MS) is not available. We conducted a retrospective analysis of eight cases of primary cutaneous nocardiosis diagnosed in the dermatology department of our hospital between 2022 and 2025, aiming to provide diagnostic insights with regard to culture, staining, mass spectrometry, 16S rRNA sequencing, and drug susceptibility testing, as well as to delineate the epidemiological characteristics of this condition.

## Introduction

*Nocardia* species are saprophytic, aerobic bacteria found worldwide in water, soil, dust, and decaying vegetation. They are opportunistic pathogens that can cause disease in humans (1, 2). More than 30 clinically relevant species within the bacterial genus *Nocardia* have been isolated from human infections. Among these, *N.asteroides* is a common cause of opportunistic infections in immunocompromised hosts (3). *N. brasiliensis*, closely following in overall clinical frequency, is the most prevalent agent of cutaneous nocardiosis (4). Cutaneous nocardiosis can be classified into two main types: primary cutaneous nocardiosis, typically resulting from direct inoculation via trauma or other local causes; and secondary cutaneous nocardiosis, which occurs due to dissemination from an internal infection, most commonly in the lungs (5, 6).

In this study, we present clinical and microbiological characteristics of 8 cases of primary cutaneous nocardiosis diagnosed in our laboratory for a period of 4 years.

## Materials and methods

We conducted a retrospective database analysis of all cases of cutaneous nocardiosis diagnosed between January 2022 and December 2025 in our mycology laboratory, which is affiliated with a dermatology-focused tertiary hospital in Hangzhou that accommodates over 1.8 million outpatient visits annually. An epidemiological questionnaire, developed specifically for this study, was administered to all enrolled patients. It encompassed the following domains: demographics (age, gender, occupation, current address, contact information), clinical presentation (disease course, history of trauma, infection type, sites and characteristics of skin lesions), laboratory findings (bacterial culture and histopathological results), underlying diseases, and other information. The study was conducted in accordance with the Declaration of Helsinki, and approved by the ethics committee of Hangzhou Third People's Hospital (protocol code:2026KA032, date of approval: 2026-01-22). Written informed consent was obtained from each participant.

Tissue fluid or pus samples were obtained via extrusion or needle aspiration. These samples were inoculated onto either Lowenstein-Jensen (LJ) medium or potato dextrose agar (PDA) and incubated at 25 °C for a minimum of 14 days to observe colony growth (Since the introduction of LJ medium in the mycology laboratory in June 2022, this medium has been consistently used for the isolation of *Nocardia*.). Initial identification of isolates was performed using Gram staining and acid-fast staining. If filamentous branching bacilli positive on Gram staining were observed, the colonies were subcultured onto LJ medium and purified for subsequent analyses, including identification by Matrix-Assisted Laser Desorption/Ionization Time-of-Flight Mass Spectrometry (MALDI-TOF MS), 16S rRNA sequencing, and bacterial drug susceptibility testing.

The subcultured isolate was then identified by MALDI-TOF MS using a Bruker Daltonik MALDI Biotyper system. Sample preparation followed an extended direct transfer method, adapted from published protocols and the manufacturer's guidelines (7), and was performed entirely within a biosafety cabinet. A fresh colony was thinly smeared onto a spot of a polished steel target plate (96-spot format). Subsequently, 1 μl of 70% formic acid was applied to the same spot and allowed to dry at room temperature. Next, 1 μl of α-cyano-4-hydroxycinnamic acid (HCCA) matrix solution was overlaid onto the dried sample. For calibration, 1 μl of Bacterial Test Standard (BTS) was applied to a dedicated quality control position on the target plate and also overlaid with 1 μl of HCCA matrix after drying. Once all spots were completely dry, the target plate was inserted into the mass spectrometer. Spectral acquisition covered a mass-to-charge (^*^m/z^*^) range of 2,000–20,000. The resulting spectral profile was analyzed using Bruker MALDI Biotyper software (version 3.1) against the standard *Nocardia* reference library (version 4.0). Identification scores were interpreted according to the manufacturer's recommended criteria. Another subculture of the isolate was submitted to Shanghai Sangong Biotech (Shanghai, China) for 16S rRNA gene sequencing, species identification was performed through a BLAST search of the GenBank database.

Susceptibilities of the *N. brasiliensis* isolates to 9 antibiotics, including aminoglycosides (amikacin, tobramycin), carbapenem (imipenem), TMP-SMX, amoxicillin, ceftriaxone, levofloxacin, linezolid, and minocycline were determined by the disk diffusion test using Gram-positive bacterial antimicrobial susceptibility testing strips (E-test method; BIO-KONT^®^, Wenzhou, China). The tests were performed and interpreted according to the guidelines of the Clinical and Laboratory Standards Institute document M2-A11 (2011) (8).

## Results

**Figure 1:**
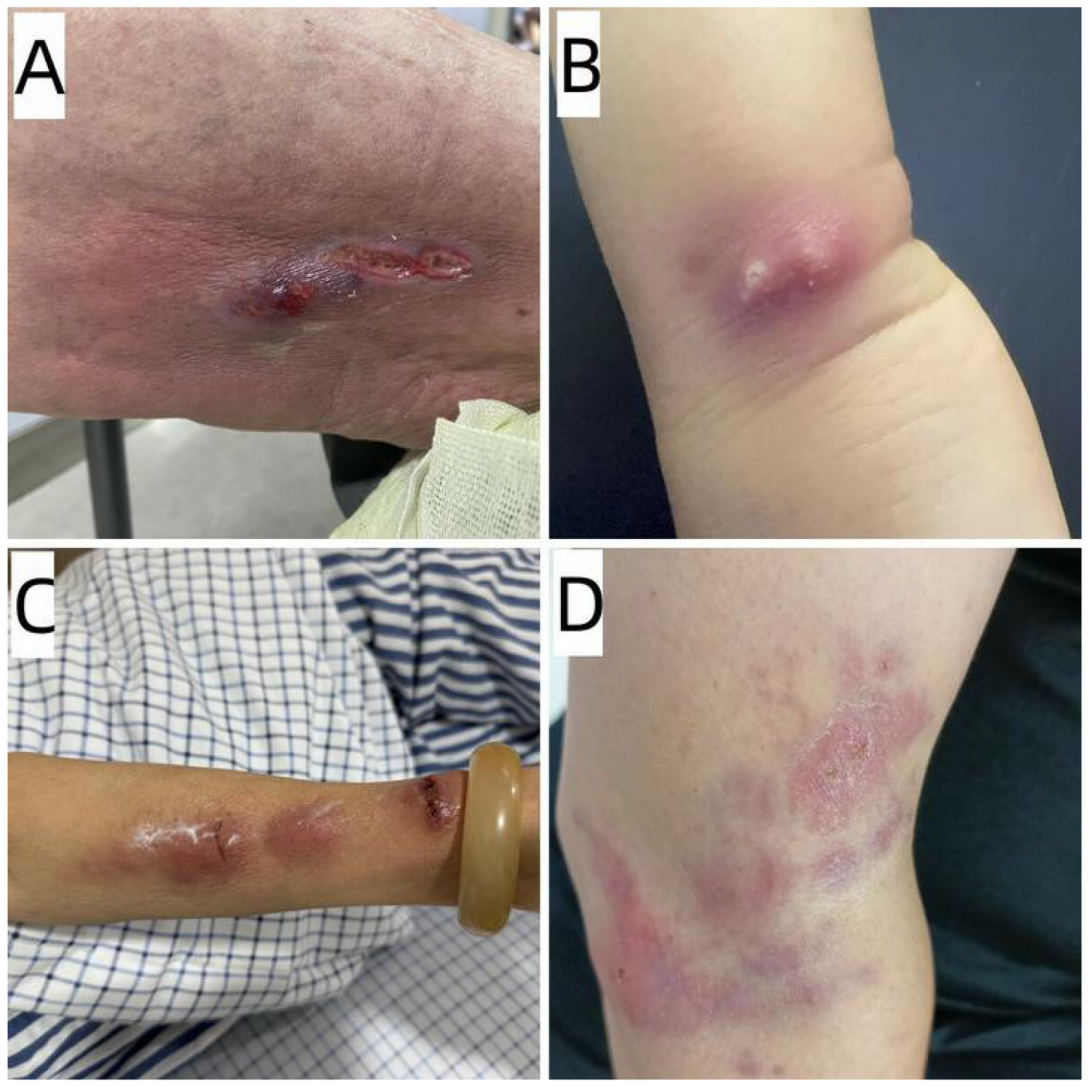
Primary cutaneous nocardiosis: **(A)** Ulcerative lesions on left thigh (patient no. 3); **(B)** Two abscesses on right wrist (patient no. 5); **(C)** Lymphocutaneous form erythematous nodules on left forearm (patient no. 4); **(D)** Erythematous plaques with mild ulcer on right arm (patient no. 8).

Table 1 summarizes the demographic and clinical characteristics of eight immunocompetent patients with cutaneous nocardiosis, with an equal gender distribution (4 males and 4 females) and an age range of 5–87 years (mean age, 57.3 years). The median duration before presentation was 7.5 days (range, 3–60 days). Only four cases reported local trauma during daily activities or exposure to environmental substances following an injury. With the exception of one patient who was comorbid with diabetes, the other seven patients reported no other underlying diseases. Imaging studies showed no lung involvement in any of the patients. All skin lesions involved the limbs, with seven cases affecting the upper extremities and only one case involving the lower extremity. Among the cases, three were characterized by ulcerative lesions (Figure 1A), three by pustules or abscesses (Figure 1B), one demonstrated painful erythematous nodules in a lymphocutaneous distribution (Figure 1C), and one showed erythema with mild ulceration (Figure 1D). All cases demonstrated the clinical signs of redness, swelling, warmth, and pain. The 8 patients were treated with various antibiotics. TMP-SMX was the antibiotic most commonly used (50.0%%), followed by intravenous infusion of ceftriaxone (37.5%). The median duration of treatment was 19.5 days (range, 7–90 days). Favorable outcome was detected in all patients.

**Table 1:** Clinical results of eight cases of Nocardia brasiliensis-induced cutaneous nocardiosis.

**Case**	**Date**	**Age(years)/ sex**	**Clinical features/site**	**Duration (d)**	**Underlying disease**	**Exposure history**	**Treatment regimen**	**Follow up/response**	**Mass spectrometry score**	**GenBank accession number**
1	2022.10	5/M	Erythematous pustule/finger	7	Without underlying disease	Wound exposure to soil	Intravenous infusion of ceftriaxone for 7 days	1 minth/cure	1.88	PX884359
2	2022.12	51/F	Painful ulcer/finger	7	Without underlying disease	Wound exposure to flour and yeast powder	Intravenous infusion of ceftriaxone for 7 days	6 month/cure	1.89	OR029249
3	2023.9	87/M	Painful ulcer/thigh	10	Diabetes mellitus	^a^Unknown	TMP-SMX for 30 days	9 months/cure	1.73	PX884361
4	2025.5	73/F	Lymphocutaneous form erythematous painful nodules/arm	3	Without underlying disease	Plant pricking injury	Intravenous infusion of ceftriaxone for 10 days	1 month/cure	1.85	PX884363
5	2025.6	63/F	erythematous painful abscesses/wrist	4	Without underlying disease	^a^Unknown	Cefaclor Sustained-release Capsules for 14 days	2 months/cure	1.83	PX884364
6	2025.6	65/M	Erythematous painful ulcer/dorsal hand	8	Without underlying disease	Insect bite	TMP-SMX for 25 days	3 months/cure	1.79	PX892072
7	2025.7	66/F	painful pustules/arm	30	Without underlying disease	^a^Unknown	TMP-SMX for 3 months	6 months/cure	1.81	PX892073
8	2025.9	49/M	Erythematous plaques with mild ulcer/arm	60	Without underlying disease	^a^Unknown	TMP-SMX for 1 months	2 month/cure	1.78	PX884365

F, female; M, male; TMP-SMX, trimethoprim–sulfamethoxazole.

^a^many potted plants in patient's house.

**Figure 2:**
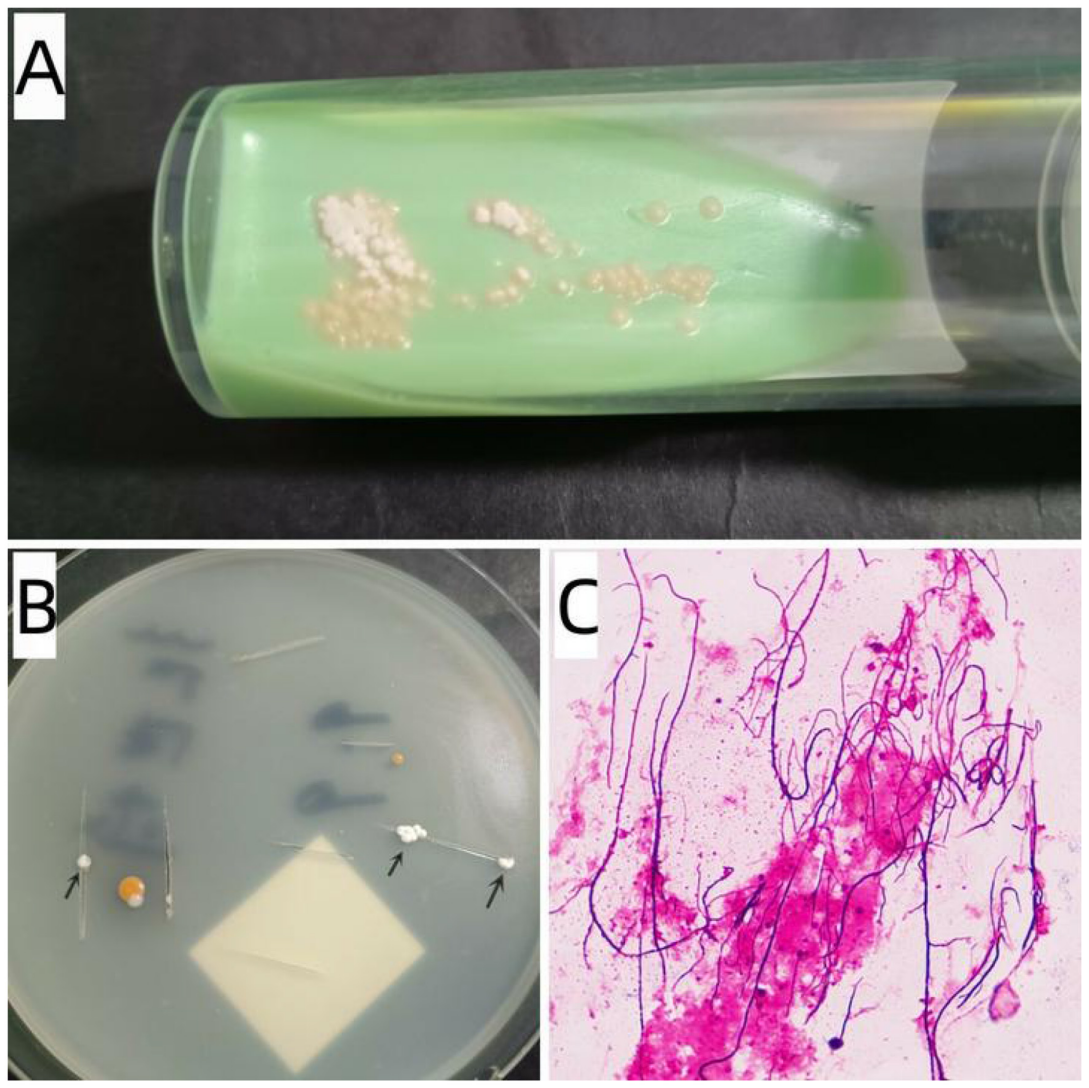
Colonies of *N. brasiliensis* grown on LJ medium **(A)** and PDA **(B)**; **(C)** Gram stain of *N. brasiliensis* colonies ( × 1000).

Visible colonies typically appeared after 5–12 days of incubation. Seven cases exhibited colonies that were pale yellow, raised, waxy, and difficult to pick on LJ medium (Figure 2A), while one case formed white, granular, firm colonies on PDA (Figure 2B). Staining characteristics generally revealed typical Gram-positive branching bacilli with partial acid-fast properties (Figure 2C). All strains were identified as *N. brasiliensis* by both MS and 16S rRNA gene sequencing, with MS scores exceeding 1.7. Subculture of Nocardia took approximately 3 days, while MALDI-TOF MS analysis required about 15 min. Reports were issued immediately upon identification. Histopathology was performed in only one case; the skin biopsy demonstrated acute suppurative inflammation containing Gram-positive branching bacilli.

Disk diffusion susceptibility testing showed that the mean MICs of the eight *N. brasiliensis* isolates were 1 μg/ml against amikacin, 2 μg/ml against amoxicillin, 32 μg/ml against ceftriaxone, 8 μg/ml against levofloxacin, 32 μg/ml against imipenem, 1 μg/ml against linezolid, 1 μg/ml against minocycline, 0.625 μg/ml against TMP-SMX, 0.125 μg/mL against tobramycin. Based on the CLSI M2-A11 interpretive criteria, all isolates were susceptible to amikacin, amoxicillin, linezolid, minocycline, TMP-SMX and tobramycin, 87.5% to ceftriaxone. Fifty percent of the isolates were susceptible to levofloxacin and imipenem.

## Discussion

*Nocardia* species are Gram-positive, partially acid-fast bacilli characterized by a beaded, filamentous, and branching morphology. More than 100 species have been identified through 16S rRNA gene sequencing (9). These organisms are ubiquitous in the environment, existing as saprophytes in soil, dust, fresh and salt water, decaying vegetation, and organic matter (3, 10). With the growing population of immunocompromised individuals, such as those with HIV/AIDS, reports of nocardial infections have increased. The prevalence of different *Nocardia* species varies by geographic region (9). Nevertheless, up to one-third of infected patients are immunocompetent, with no underlying diseases (3, 10, 11). which was confirmed in our study. Human infection is exogenous, occurring either through inhalation—leading to pulmonary disease—or via direct inoculation into the skin and soft tissues. The clinical spectrum primarily includes pulmonary, cutaneous/soft tissue, and cerebral infections (12).

Patients with cutaneous nocardiosis frequently report a history of local trauma, such as puncture wounds, insect bites, abrasions, or cat scratches, even if many cannot recall a specific injurious event (13). Our study showed that half of the patients had a history of trauma, while the remaining half had an unknown exposure history. Notably, all four patients with an unreported exposure history kept numerous potted plants at home, This may be related to inadvertent exposure during routine gardening activities. Spiliopoulou et al. reported that gardening-related injuries and insect bites are the most common forms of trauma history in cases of cutaneous nocardiosis (14). Currently, *N. brasiliensis* accounts for the majority of cutaneous nocardiosis cases (5, 10, 15).

It has been reported that primary cutaneous nocardiosis can rarely become disseminated (16–18). When the cutaneous barrier is disrupted, *Nocardia* can invade deeper tissues, triggering an acute inflammatory response and leading to abscess formation. Initially, such lesions may be clinically indistinguishable from those caused by other microorganisms, such as *staphylococci* or *streptococci*, potentially resulting in misdiagnosis (19). Cutaneous infections caused by *Nocardia* species typically follow an indolent clinical course (3). However, our study demonstrated that the median duration of illness among patients was just over 7 days.

Cutaneous nocardiosis can mimic various conditions. Its clinical presentations include lymphocutaneous (sporotrichoid) infection, actinomycetoma, superficial skin lesions (such as pustules, ulcers, granulomas, abscesses, or cellulitis), or secondary infection resulting from hematogenous dissemination (20). Clinically, primary cutaneous nocardiosis is classified into two main forms: an acute type (presenting as superficial skin/soft tissue or lymphocutaneous infection) and a chronic type (mycetoma). Initially, pustular lesions may develop, potentially progressing to a spectrum of conditions including cellulitis, abscesses, ulcerative or sporotrichoid lesions, and linear/keloid-like lesions or granulomas (21). The main differential diagnoses include sporotrichosis, infections with atypical mycobacteria (*Mycobacterium marinum* and *M. chelonae*), leishmaniasis (caused by *Leishmania braziliensis*), and tuberculosis (22). Nocardiosis is an infrequently encountered condition in routine clinical practice. However, as it may resolve completely with antibiotic therapy in some patients, it is plausible that cases are occasionally treated under the diagnosis of phlegmon or other conditions. The possibility of nocardiosis should be considered in patients who present with redness, swelling, or similar symptoms following the initial improvement of post-traumatic skin lesions (5). The manifestation of pain, redness, swelling, and heat in most cases of cutaneous nocardiosis, as shown in our study, could be valuable in clinically differentiating this disease from the other conditions under consideration.

Primary cutaneous nocardiosis poses a diagnostic challenge due to its nonspecific presentation. It should be considered in the differential diagnosis of small pustular lesions, which may progress to overt suppurative inflammation (9). Definitive diagnosis relies on tissue culture (23). *Nocardia* grows on most standard media for bacteria, fungi, or mycobacteria, typically within 2–5 days, although growth can be slow, requiring incubation for up to 14–21 days (24). Therefore, close communication with the laboratory and prolonged culture periods of at least 10 days are essential for proper detection (9). It is worth noting that *N. brasiliensis* grows well on both LJ medium and PDA, with colonies appearing creamy-white and firm on these media, indicating that both can be used for the isolation of *N. brasiliensis* (14).

Accurate identification of *Nocardia* isolates using phenotypic and genotypic methods is important for delineating the disease spectrum associated with each species, elucidating epidemiological patterns, and predicting antimicrobial susceptibility—all of which are essential to guide appropriate treatment (12). The gold standard for identifying *Nocardia* species relies on molecular methods involving amplification and sequencing of one or more target genes, such as 16S rRNA, hsp65, secA1, and sodA (14). MALDI-TOF MS has emerged as a reliable alternative, enabling rapid, cost-effective, and accurate species-level identification of *Nocardia* (25). This technique achieves correct identification in 94%−100% of cases for most species and is being increasingly adopted in routine practice (24). Our study confirmed that MALDI-TOF MS identification of Nocardia species showed complete concordance with the results obtained by 16S rRNA sequencing.

Empiric therapy for nocardiosis relies primarily on TMP-SMX, to which more than 90% of clinical isolates are susceptible (26–28). In immunocompetent patients with cutaneous disease, TMP-SMX monotherapy is frequently adequate. For those with contraindications (e.g., allergy), alternative regimens include linezolid, amikacin, minocycline, moxifloxacin, or amoxicillin-clavulanic acid (12). We found that intravenous ceftriaxone may serve as an effective alternative for the treatment of localized cutaneous nocardiosis (29), a finding that warrants further clinical validation.

In conclusion, the diagnosis of cutaneous nocardiosis should be based on a combination of environmental exposure history, clinical presentation (typically characterized by acute or subacute inflammatory manifestations), and microbiological examination. *Nocardia* can grow on various routine isolation media, particularly Löwenstein-Jensen (LJ) medium. MALDI-TOF MS has emerged as a reliable alternative, enabling rapid, cost-effective, and accurate species-level identification of *Nocardia*. As an empirical treatment, intravenous ceftriaxone may serve as a viable alternative therapeutic option for localized cutaneous nocardiosis. A key limitation of this study is its relatively small sample size and the fact that all patients were enrolled from a single center. Consequently, the epidemiological findings related to cutaneous nocardiosis should be interpreted with caution and may not be generalizable to the broader national population. to the corresponding author.

## Data Availability

The original contributions presented in the study are included in the article/supplementary material, further inquiries can be directed to the corresponding author.
